# Exploring Patient Experience of Chest Pain Before and After Implementation of an Early Rule-Out Pathway for Myocardial Infarction: A Qualitative Study

**DOI:** 10.1016/j.annemergmed.2019.11.012

**Published:** 2020-04

**Authors:** Amy V. Ferry, Fiona E. Strachan, Stacey D. Stewart, Lucy Marshall, Kuan K. Lee, Atul Anand, Anoop S.V. Shah, Andrew R. Chapman, Nicholas L. Mills, Sarah Cunningham-Burley

**Affiliations:** a BHF Centre for Cardiovascular Science, University of Edinburgh, Edinburgh, United Kingdom; b Usher Institute of Population Health Science and Informatics, University of Edinburgh, Edinburgh, United Kingdom; c Centre for Biomedicine, Self and Society, University of Edinburgh, Edinburgh, United Kingdom

## Abstract

**Study objective:**

High-sensitivity cardiac troponin assays enable myocardial infarction to be excluded in the emergency department (ED). As part of a prospective clinical trial, we explore how introducing an early rule-out pathway may affect patient experience of chest pain.

**Methods:**

In a qualitative study, participants presenting to the ED with suspected acute coronary syndrome, and for whom the diagnosis of myocardial infarction was excluded, were interviewed before (n=23) or after (n=26) implementation of an early rule-out pathway. Preimplementation, diagnosis of myocardial infarction was excluded on serial troponin testing requiring admission to the hospital. Postimplementation, diagnosis could be excluded in the ED, enabling direct patient discharge. Semistructured interviews exploring the patients’ illness experience were conducted approximately 1 week postdischarge, transcribed verbatim, and analyzed thematically. Themes emerging pre- and postimplementation are described.

**Results:**

Common themes emerged across both pathways: participants commonly sought health care advice before presenting to the ED; a discordance may exist between the objective interpretation of troponin results by clinicians and the patients’ experience of illness; and pretest information, trust in the clinician, and active listening may enhance reassurance gained from negative test results. Other themes related to the care pathway were that routine care procedures appeared to be a source of frustration for participants requiring hospital admission, and patients assessed with the early rule-out pathway appeared less likely to appraise their future health status.

**Conclusion:**

The early rule-out of myocardial infarction may be enhanced by recognition of patient out-of-hospital experience and improved communication surrounding reassurance and future cardiovascular health goals.

## Introduction

### Background

Patients with suspected acute coronary syndrome are responsible for 6% of emergency department (ED) presentations.^1^ The majority of these patients will not receive a diagnosis of myocardial infarction,^2^ but clinical guidelines have recommended serial cardiac troponin-level testing to safely rule out the diagnosis, which often requires admission to the hospital.^3^^,^^4^ Because EDs are under increasing pressure to reduce the number of patients admitted to the hospital,^5^ the use of high-sensitivity troponin assays to exclude myocardial infarction earlier or at presentation may increase efficiency in the ED setting.

**Editor’s Capsule Summary**

*What is already known on this topic*

Some emergency departments (EDs) are using rapid myocardial infarction rule-out pathways to avoid hospitalization of patients with potential acute coronary syndrome.

*What question this study addressed*

This qualitative study assessed patient impressions before and after implementation of an ED rapid rule-out pathway. Forty-nine patients were interviewed 1 week postdischarge and 5 major themes emerged across both pathways.

*What this study adds to our knowledge*

A discordance may emerge between physician relief at the absence of acute coronary syndrome according to normal troponin levels and patient concern for continued unexplained chest symptoms. Patients may also be less likely to assess their health behaviors and risk of future cardiovascular disease.

*How this is relevant to clinical practice*

Understanding patient perceptions may help physicians provide better care for ED patients with similar diagnostic trajectories.

### Importance

The European Society of Cardiology and the National Institute for Health and Care Excellence have endorsed early rule-out pathways based on high-sensitivity cardiac troponin assays.^6^^,^^7^ The Food and Drug Administration has now also approved high-sensitivity cardiac troponin assays for clinical use. A number of strategies have been proposed to identify patients at presentation or 1 to 2 hours after presentation who may be suitable for discharge directly from the ED.^8^, ^9^, ^10^, ^11^, ^12^, ^13^, ^14^ Although the adoption of these rule-out pathways could improve efficiency in the ED and therefore lead to major benefits for health care providers, patients will spend less time within the health care setting and may have fewer assessments from specialists and opportunities to discuss the nature of their symptoms, and as a consequence may be less likely to be reassured their symptoms are benign. Previous research into the experience of patients with acute chest pain who present to the ED has shown that they may be discharged with unanswered questions,^15^ feelings of uncertainty,^16^ and the need to feel more supported after discharge.^17^

### Goals of This Investigation

In this qualitative study embedded into a prospective clinical trial,^18^ we aimed to explore the experience of 2 groups of patients undergoing assessment for suspected acute coronary syndrome before and after implementation of an early rule-out pathway for myocardial infarction to identify how the assessment pathway affects the patients’ experience. The insights revealed will ensure that early rule-out pathways can be applied to patient assessment in a way that responds to patients’ needs.

## Materials and Methods

### Study Design and Setting

This qualitative study was embedded into a clinical trial evaluating the safety and efficacy of an early rule-out pathway for myocardial infarction across secondary and tertiary care hospitals in Scotland.^18^ The qualitative component was conducted at the lead site in the ED of the Royal Infirmary of Edinburgh, a tertiary care hospital. The clinical trial was approved by the national research ethics committee and conducted in accordance with the Declaration of Helsinki. This qualitative substudy was prespecified in the trial protocol.

### Selection of Participants

Patients with suspected acute coronary syndrome were recruited from the ED between March 2015 and June 2017. Patients older than 18 years, for whom the attending clinician requested cardiac troponin-level testing for suspected acute coronary syndrome, and who were discharged on the basis of a negative evaluation result for myocardial infarction were eligible for inclusion. Eligibility was verified with the electronic patient record. The main trial recruited consecutive patients, but the substudy used purposive sampling to ensure representation across age and sex categories (male patients, female patients, >65 years, and ≤65 years). In a clinical scenario, the pathway is applied nonselectively to any patient presenting with symptoms suggestive of acute coronary syndrome. It was therefore thought important that a broad spectrum of patients be recruited to this study; therefore, participants were not stratified further. Sampling and recruitment occurred deliberately slowly to allow concurrent data collection and analysis.

**Figure 1:**
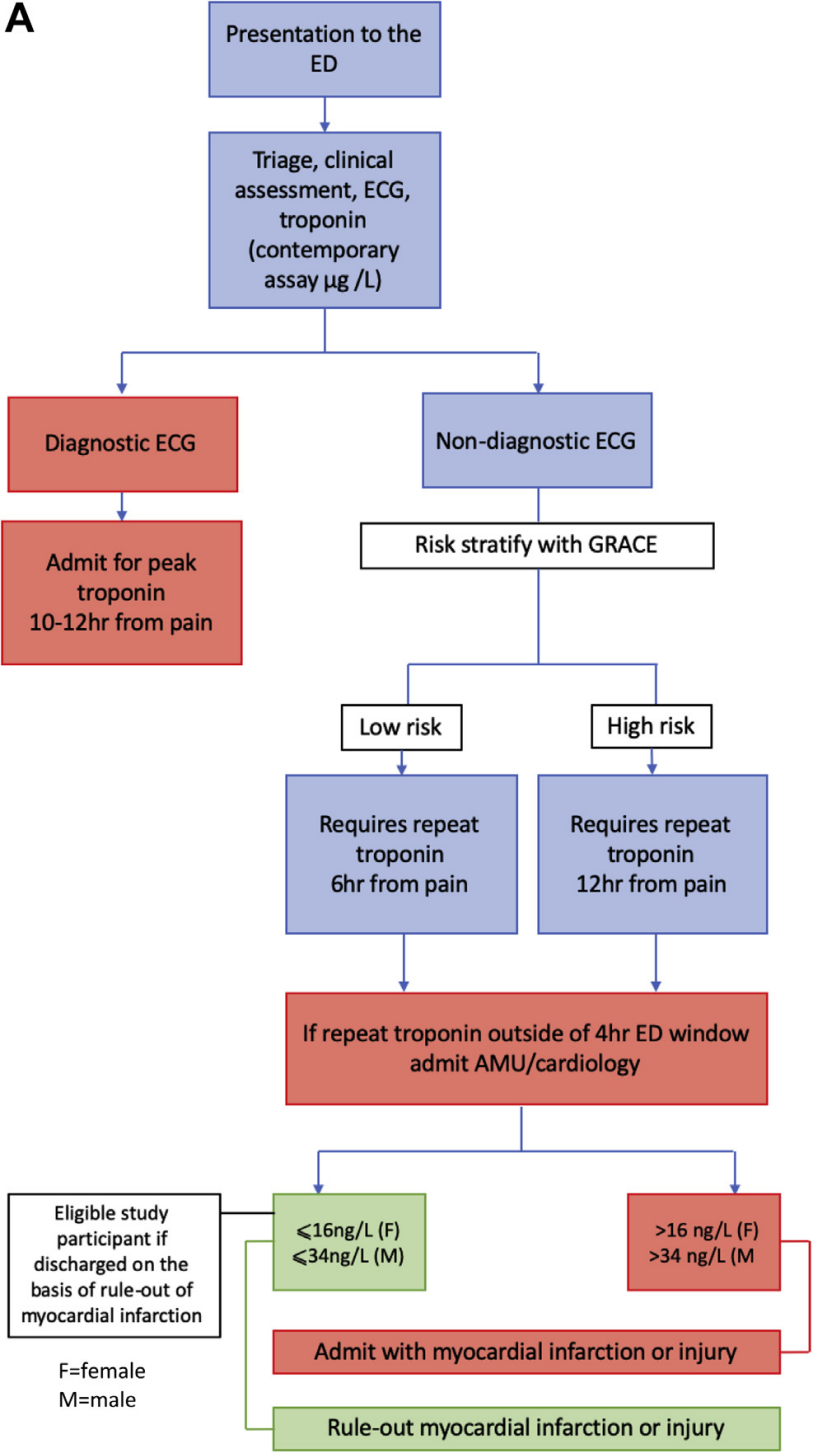

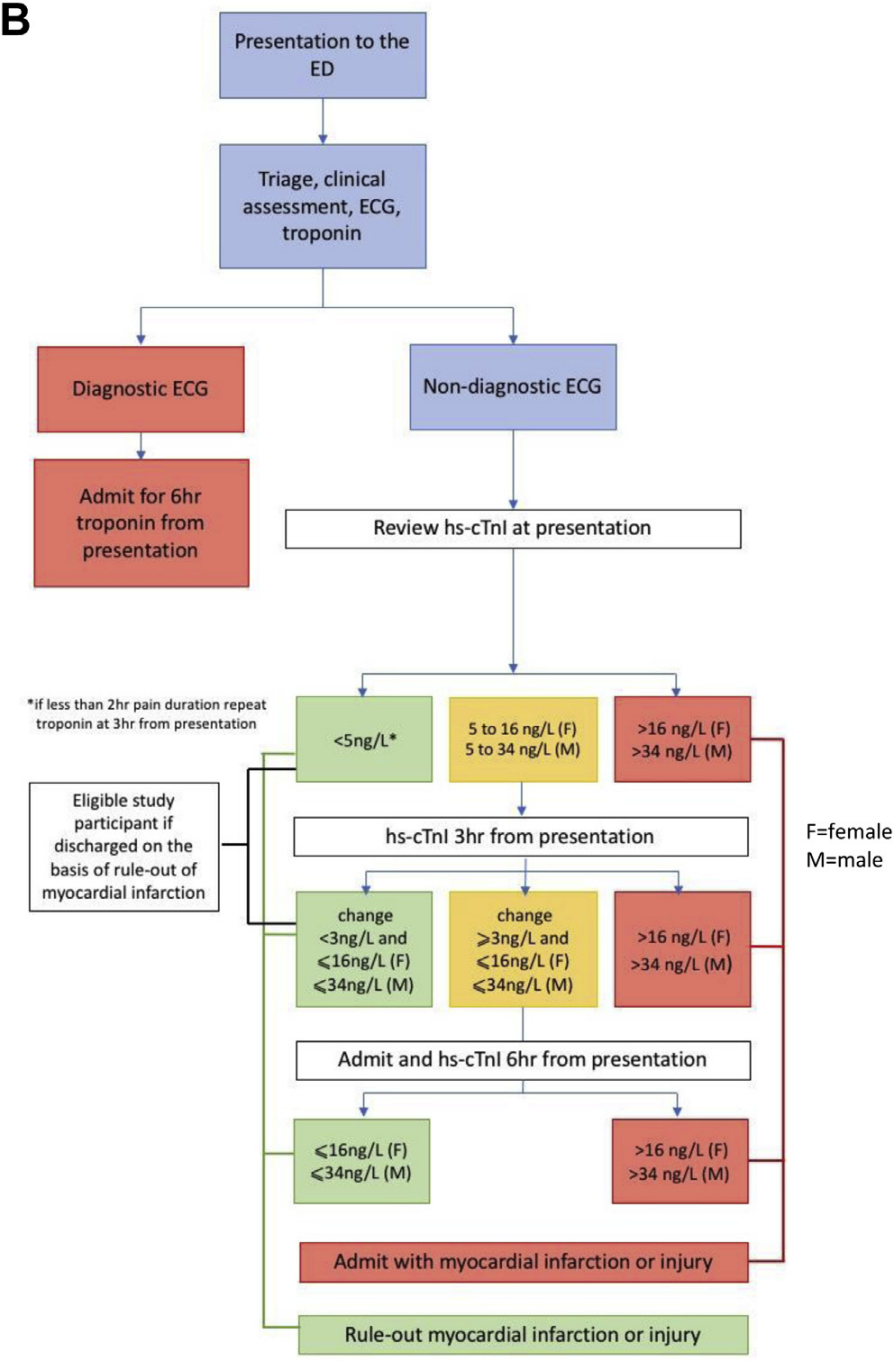
Summary of chest pain pathway and identification of eligible participants. *A*, Before implementation of the early rule-out pathway. *B*, After implementation of the early rule-out pathway. *GRACE*, Global Registry of Acute Coronary Events; *AMU*, acute medical unit.

The early rule-out pathway was implemented on February 15, 2016. Before this date, patients were admitted to a medical assessment unit or cardiology ward for serial troponin-level testing to determine peak troponin level at 10 to 12 hours after symptom onset. Eligible participants were those discharged in accordance with negative serial test results for myocardial infarction. Follow-up care was at the discretion of the assessing clinician. After this date, myocardial infarction was excluded in accordance with troponin concentration at presentation or at 3 hours, enabling patients to be discharged directly from the ED.^19^ Follow-up care remained at the discretion of the assessing clinician. The postdischarge care of patients with the rule-out of myocardial infarction was not altered by the implementation of the early rule-out pathway. Identification of study participants according to pathway is shown in Figure 1*A* and *B*.

The early rule-out pathway was developed in a substudy of patients presenting with suspected acute coronary syndrome between June 1, 2013, and September 30, 2015. Blood samples were obtained at presentation and at 6 to 12 hours after symptoms onset for high-sensitivity cardiac troponin-level testing as part of routine clinical care. Patients provided informed consent to obtain an additional blood sample at 3 hours as previously described.^19^^,^^20^ The troponin assay used was the Abbott ARCHITECT_*STAT*_ high-sensitivity cardiac troponin I assay (Abbott Laboratories, Abbott Park, IL).^21^ The upper reference limit 99th centiles were determined in 4,590 samples from healthy individuals as 16 ng/L for women and 34 ng/L in men.^22^

### Data Collection and Processing

Participants were approached by A.V.F. or an ED research nurse with an information sheet while in the ED or medical assessment unit (for those recruited in the preimplementation phase). Participants were contacted by telephone at least 24 hours after discharge to discuss participation and arrange a date for interview 1 week postdischarge if appropriate to do so. After written informed consent was gained, interviews were conducted in a place of the participants’ choosing, including their own home (16 pre- and 21 postimplementation), the hospital (5 pre- and 2 postimplementation), or a private meeting room at their workplace (2 pre- and 3 postimplementation). For the majority of interviews, only the interviewer and participant were present, although a family member was present during 5 interviews.

A research diary was kept to document reflections after each interview, and regular debriefing with a supervisor (S.C.-B.) was used to promote researcher reflexivity.^23^ This also served as a decision trail to demonstrate how interpretative analysis evolved during the study and therefore increased the trustworthiness of the study.^24^ Interviews lasted between 18 and 88 minutes. Data collection and analysis occurred concurrently. Recruitment continued until saturation was achieved and additional interviews did not yield new insights.^25^

A female cardiology research nurse with experience in qualitative interviewing techniques (A.V.F.) conducted semistructured, detailed interviews, using a topic guide developed from a literature review and clinical experience of the study team (Appendix E1, available online at http://www.annemergmed.com). The interviews proceeded as a guided conversation, ensuring the same range of topics was covered yet allowing respondents flexibility in how they answered and in introducing new issues as relevant to them. A.V.F. was not involved in the clinical care of patients and was introduced to participants as a researcher. If participants questioned the interviewer directly, her identity as a nurse was revealed. Interviews all started with the same opening question: “Could you tell me what happened to take you into [the] hospital last week?” Interviews were audio recorded and transcribed verbatim by a professional transcription company. Each transcript was checked for accuracy against the audio file by A.V.F. Data are presented as quotes from transcripts, with “I” prefixing interviewer speech and “P” participant speech.

### Primary Data Analysis

**Figure 2:**
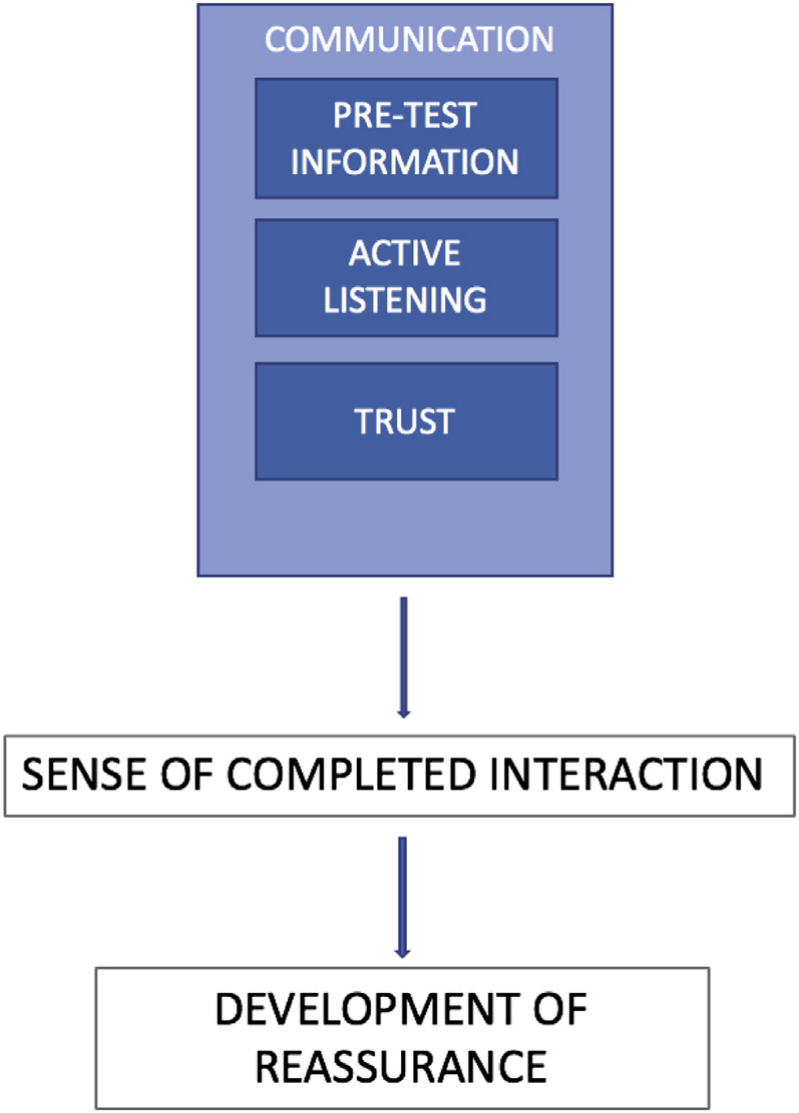
Communication interventions aiding the development of reassurance.

This study was guided by broadly phenomenological principles,^26^ aiming to uncover the meaning and relevance of experience. An interpretive approach was used to analyze data thematically,^27^ using abductive reasoning, which seeks to identify meaning from the accounts in iteration with previous knowledge from the field.^28^ This involves repeatedly reading and interpreting accounts to search for patterns of meaning in the data. Data were coded by A.V.F. under the guidance of an experienced senior qualitative researcher (S.C.-B.). Interviews were read multiple times, and relationships between codes were explored to identify themes that were derived from the data. The grouping of codes into themes was performed through discussion with S.C.-B., including review of the patient narratives and coding categories. Through this process, the themes and model were created (Figure 2). Themes arising within patient accounts from both pathways were compared to search for similarities and differences in which the chest pain pathway may be implicated. Data were managed with NVivo (version 10; QSR International, Victoria, Australia). When clear differences between care pathways were observed, the results were quantified. When themes were pervasive across both pathways, quantification of results did not add value to the analysis.

## Results

### Characteristics of Study Subjects

**Figure 3:**
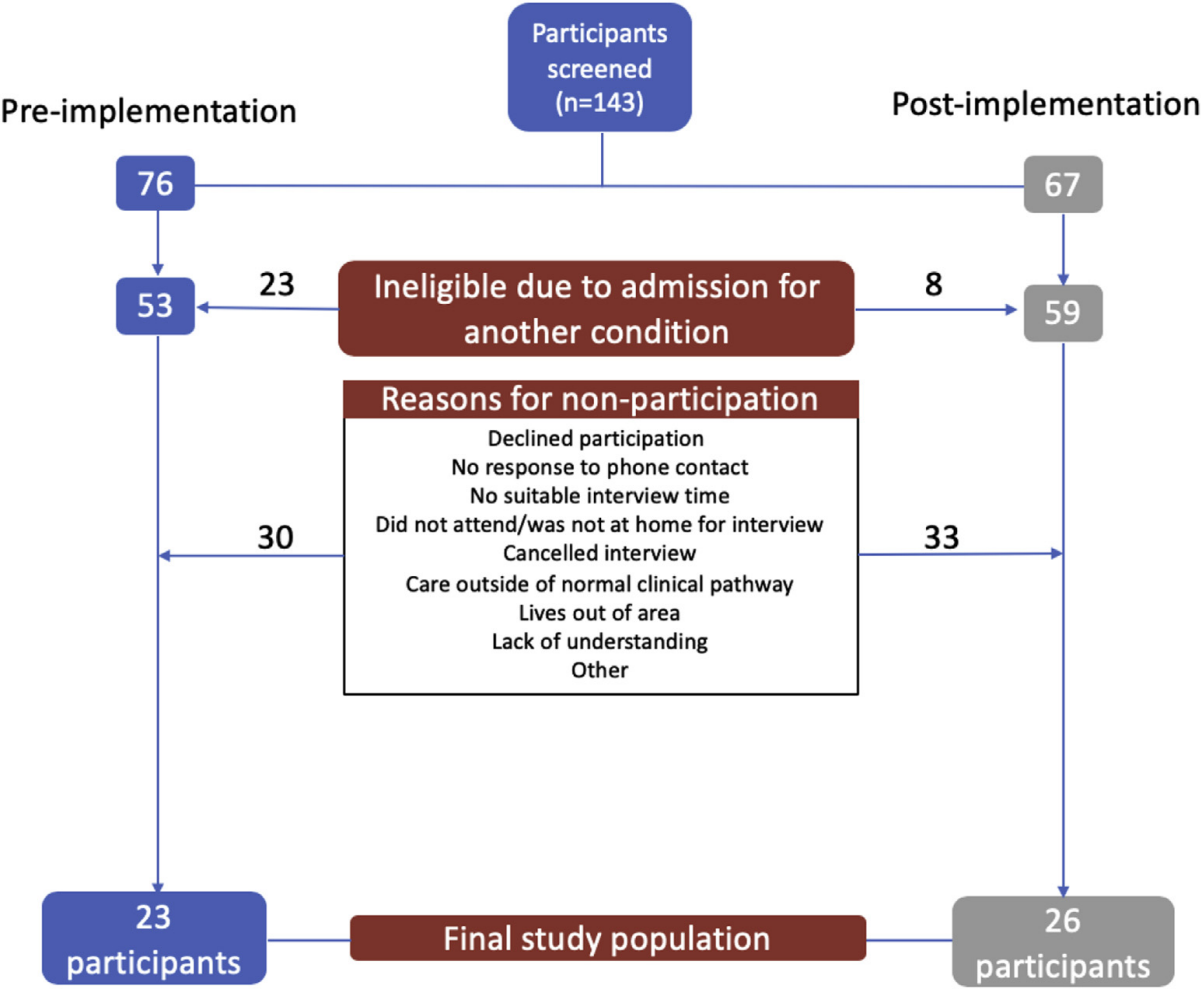
Reasons for nonparticipation of potential study participants.

One hundred forty-three patients were approached with an information sheet; 23 participants were interviewed before and 26 participants after implementation of the early rule-out pathway. Reasons for nonparticipation are outlined in Figure 3. The mean age of men was 59 years (SD 14 years) preimplementation and 60 years (SD 15 years) postimplementation, and for women it was 58 years (SD 15 years) preimplementation and 61 years (SD 15 years) postimplementation. The median length of hospital stay was 10.5 hours (interquartile range 8.2 to 12.3 hours) and 3.4 hours (interquartile range 2.5 to 3.9 hours) before and after implementation of the early rule-out pathway, respectively. Further details of study participants are reported in Table 1.

**Table 1:** Baseline characteristics of study population.

	All	Preimplementation	Postimplementation
**Study sample**			
Participants, No. (% women)	49 (45)	23 (39)	26 (50)
Women >65 y, No. (%)	12 (25)	4 (18)	8 (31)
Women ≤65 y, No. (%)	10 (20)	5 (22)	5 (19)
Men >65 y, No. (%)	16 (33)	7 (30)	9 (34)
Men ≤65 y, No. (%)	11 (22)	7 (30)	4 (16)
**Men, age, y**			
Mean (SD)	60 (14.4)	59 (4.4)	60 (14.9)
Range	20–85	33–85	20–83
**Women, age, y**			
Mean (SD)	59 (14.6)	58 (15.2)	61 (15.3)
Range	35–82	35–72	41–82
**Medical history, No. (%)**			
Smoking	20 (41)	11 (48)	9 (35)
Diabetes mellitus	6 (12)	4 (17)	2 (8)
Hypertension	22 (45)	9 (39)	13 (50)
Hyperlipidemia	19 (39)	9 (39)	10 (38)
Family history	25 (51)	14 (61)	11 (42)
Angina	11 (22)	5 (22)	6 (23)
Myocardial infarction	15 (31)	9 (39)	6 (21)
Previous PCI	11 (22)	7 (30)	4 (15)
Previous CABG	2 (4)	1 (4)	1 (4)
Heart failure	0	0	0
Cerebrovascular disease	0	0	0
Peripheral vascular disease	1 (2)	0	1 (4)
Length of stay, median (interquartile range), h	—∗	10.5 (8.2–12.3)	3.4 (2.5–3.9)

*PCI*, Percutaneous intervention; *CABG*, coronary artery bypass graft.

∗ Dash indicates value not calculated

### Main Results

Five important concepts could be identified in the accounts of participants interviewed both before and after implementation of the early rule-out pathway. Where variance in response was noted between pathways, these differences have been highlighted.

First, it was rare for a patient to make the decision to attend the hospital for assessment independently without previous contact with the health service. Participants revealed a careful consideration about their personal justification to use the health service, resulting in their seeking confirmation that their symptoms warranted professional health care assessment. Only 7 of 49 patients attended the ED without consulting a second party. Many patients (61% [30/49]) had sought advice from a general practitioner or NHS 24 (a telephone advice and triage service that runs out of hours in Scotland), with the recommendation to attend the hospital for assessment, or the even stronger message of “telephone 999” (the equivalent of 911 in the United States). The remainder of patients consulted lay networks for advice. It was also apparent in several transcripts that receiving advice from a general practitioner or NHS 24 call handler to attend the ED for assessment may actually strengthen patients’ belief that their symptoms are serious. This is illustrated through participant quotes in Table 2.

**Table 2:** Evidence for themes of help seeking behavior, discordance, and reassurance.

Theme	Evidence	Participant
Help-seeking behavior	I: So why NHS 24? P: That was…I don’t know. That was…to me, that’s a step before you phone 999 for an ambulance. Erm, and then you’re giving somebody else the decision to… I: Yes. P: …[I]f, though…if they think it’s serious enough, then I, I…I’m wary about taking up people’s time, et cetera…. I: Okay. P: …[I]f it’s, you know, not warranted, sort o’ thing.	Participant 19, >65 y, woman (preimplementation)
	P: Well, it’s, it was certainly, um, again, I mean, it was one of these situations where, you know, I had to phone up and the receptionist said, “Is it an emergency,” and I said, “Well, all I can tell you is this is what’s happened….” I: Yeah. P: …[T]his has been my experience, and she said…so she made the de-decision, the receptionist made the decision…[to telephone an ambulance]. I: Right. P: …[W]hich was great because I didn’t want to be wasting the, the GP’s [general practitioner’s] time….	Participant 38, ≤65 y, man (postimplementation)
	I: So when you got the advice to attend A&E… P: Mm-hmm. I: …[H]ow did you feel about that? P: Oh, that…that…it…it then went from “I’ve got a pain in my chest” to “I’ve got a pain in my chest and someone thinks that my symptoms are serious.” I: Right. P: Um, so it…it escalated a wee bit. So I went from thinking, well, maybe there’s something wrong to, well, it’s maybe more than a maybe.	Participant 49, >65 y, man (postimplementation)
Discordance	I: I’d like to explore the reason why you don’t feel you can go out. P: I just thought…I think…I just thought I got such a fright, really… I don’t know if I still thought I could see me having a heart attack….	Participant 5, >65 y, woman (preimplementation)
	P: At the time they told me I could go home, that felt good…. I know there’s a bigger picture.	Participant 11, ≤65 y, man (preimplementation)
	P: I just know there is something going on. Whether it’s my heart or not I don’t know.	Participant 38, ≤65 y, man (postimplementation)
	P: At the back of my mind you’re thinking is this my heart? Am I going to have a heart attack?	Participant 48, ≤65 y, woman (postimplementation)
Reassurance	P: There was absolutely no damage to my heart.	Participant 2, >65 y, woman (preimplementation)
	P: I feel quite confident about my heart now because of the tests. I’ve no concerns about my heart.	Participant 15, >65 y, man (preimplementation)
	P: Once I was discharged with a clean bill, I parked it.	Participant 24, ≤65 y, man (postimplementation)

Second, and also common to participants assessed both pre- and postimplementation of the early rule-out pathway, a discordance between the objective interpretation of a troponin concentration by a clinician and the ongoing illness episode experienced by the patient was illustrated. Patient accounts revealed that, although a medical consultation may have concluded for the clinician with a negative evaluation result for acute coronary syndrome and a perception that reassurance had been provided, for some patients, their illness episode was still very much ongoing at the subsequent interview, whichever clinical pathway they had followed. A discordance was therefore found to exist between the objective interpretation of a troponin value by the clinician and the significance that result held to the patient in the context of his or her illness experience. When the pathway, driven by the high negative predictive value of a low troponin concentration, reassured the clinician that the patient did not warrant further investigation (inferred by the decision to discharge the patient), for some patients, this reassurance was not perceived despite clinical notes stating that reassurance had been given. Some patients left the hospital without a satisfactory conclusion to their illness episode, with ongoing questions about the cause of their pain and some patients still believing that their pain may have had a cardiac substrate. Participant quotes provide illustrations in Table 2.

Third, common to both pathways and suffusing the interviews, was the theme of “reassurance,” which appeared both implicitly and explicitly. When discordance as described above was present, reassurance appeared much more difficult to achieve. An initial code of “completed interaction,” signifying that the patient perceived that the health care encounter had come to an end, was used to conceptualize the meaning of reassurance in a clinical context. Data arising from the interviews suggest that reassurance is a process that has to be built atop certain foundations laid during the clinical assessment. Reassurance was more apparent within patient accounts if an alternative diagnosis about the cause of the chest pain was offered, if the participant was referred for outpatient investigations signaling ongoing care, or if the participant had a very low level of concern that the pain may have had a cardiac cause. For participants who did believe their pain could be cardiac in nature, the development of reassurance was influenced by 3 contributing factors: timing of information giving, patient-clinician interaction, and the development of trust. These factors all involve effective communication between the patient and clinician. Providing information about troponin testing and the possible meaning of results before actual testing appeared to relate to positive expressions of reassurance within interviews. Evidence of the clinician’s using active listening that acknowledged the patients concerns also enabled trust in the clinician to develop. When the factors of pretest information, active listening, and trust were satisfied, participant perception of closure of the acute illness episode could be observed in interviews. This model is presented in Figure 2. Participant quotes provide illustrations in Table 2.

The standard assessment procedures carried out by hospital staff could be interpreted by patients in a manner different from that intended. The efficiency and speed with which ED staff carried out initial assessment procedures were interpreted by some participants as confirmation that their symptoms were a cause for concern. Likewise, the routine nature of repeated blood sampling for peak troponin level as required by the preimplementation pathway could also be interpreted by participants to signify a higher likelihood that their symptoms may have been due to myocardial infarction. Additionally, some participants spoke in terms of being part of a “process.” For some participants, the routine nature of the assessment process was evidence that clinicians would perform appropriate actions because they were following a protocol. For others, the protocol-driven actions were interpreted as lack of personalized care.

Expressions of negativity concerning ambiguity in regard to an overnight admission to the hospital and the need to repeat a symptom history to multiple practitioners were common in interviews of participants assessed before implementation of the early rule-out pathway (78%; 18/23) but less so in those assessed after its implementation (15%; 4/26). These interpretations are illustrated through participant quotes in Table 3.

**Table 3:** Evidence for themes of influence of hospital routines and approaches to future health.

Theme	Evidence	Participant
Influence of hospital routines	P: The only thing that I’ve found a bit irritating was the inconsistency of the doctors when I got in there. You’re going home. You’re staying in. You’re going home. You’re staying in. So there was 3 different doctors, told me different things.	Participant 4, >65 y, woman (preimplementation)
	P: It was frustrating, you know, to have to tell the nurse what had happened, and then frustrating to have to tell someone else what had happened, and then a doctor what had happened, and then the consultant what had happened; you know what I mean. So there was, I was thinking, Jesus, can we not just get everybody in the room, and I'll tell you, look, here is what happened, guys.	Participant 23, ≤65 y, man (preimplementation)
	P: And, uh, then a lady came back and then she said I needed to take an aspirin and I would need to stay in till after 12 to get another blood test, because it...she said if it was the heart and any damage had been done, this test showed up something that’s released into the blood. Um, and then I thought, oh, no. Then it was...slight panic set in, because I thought, it’s not as straightforward as I thought. What if they’ve found something?	Participant 14, >65 y, man (preimplementation)
	P: If, if the emergency department are going through their protocols, then clearly that’s their protocols for, for that. If, however, there is, something that’s flagging up, then I think it should either be referred back to the GP [general practitioner] to take up…or sent to whoever needs to make, you know, the decisionmaker. I think not being listened to is critical.	Participant 38, ≤65 y, man (postimplementation)
Approaches to future health	P: Now, for me, I would have said, I’m 53, history of…aged myself prematurely here…um, you know, cardiac problem history in the family, overweight, don’t smoke and things, so those are the risk factors, aren’t they? But I would have probably seen that as an opportunity to say, okay, you’ve maybe had a bit of a scare here; these are the things you should look out for if this happens again. Because there was none of that advice, in terms of, right, if…this is what you…so if this pain happens again, that’s okay, ’cause that’s just your frozen shoulder, but these are the, the warning signs you should maybe look out for, or these are the things you should be doing to reduce your risk of heart…problems, or even go and see your GP [general practitioner] for a general checkup….	Participant 20, ≤65 y, woman (preimplementation)
	P: Well, it wasn't a heart attack this time. Will there be a heart attack next time? You know, that, that's my concern. Um, I need to change some lifestyle things which I know about, and I will, I am. Um, but I need to also get to the, the root cause of the stress, and anxiety bit, which is work related.	Participant 11, ≤65 y, man (preimplementation)

The final theme, and where a further difference was observed between chest pain pathways, was the way in which participants made use of their acute chest pain presentation to the hospital as an opportunity to consider their future heart health. “Approaches to future health” was an unelicited theme within interview transcripts. Participants demonstrating an awareness of future heart health did so in 3 main ways. First, they discussed their incentive to modify their lifestyle as a result of an acute chest pain admission. Second, some participants suggested their acute chest pain presentation and assessment was an appropriate opportunity for health promotion activities. Third, some participants discussed how the rule-out of myocardial infarction related to their overall heart health and their future susceptibility to heart disease. Some patients made no reference to their future health during the course of the interview. This analysis has consequently revealed 3 possible perspectives by which participants may relate to their future health status. For some participants, continuing good health was taken for granted and therefore did not have particular salience in their everyday lives. For others, the way in which they reacted to the chest pain episode varied in accordance with their position in the adult life course and their current health status. For example, their current health status because of comorbidities appeared to have dominance over the acute chest pain episode, leading to discourses of fatalism and certainty of future ill health. Other participants used the chest pain presentation, and therefore the recognition of a physical manifestation of ill health, as a trigger to appraise health behaviors and assess their future risk of cardiovascular disease. This aspect was more commonly observed in patients interviewed before implementation of the early rule-out pathway (43% [10/23] preimplementation versus 19% [5/26] postimplementation). Participant quotes provide illustrations in Table 3. Additional illustrations for all themes are shown in Table E1 (available online at http://www.annemergmed.com).

## Limitations

Our sample was limited to patients assessed in a single ED in Scotland and therefore may not represent the views of more diverse populations, particularly in regard to ethnicity. One of the main reasons for nonparticipation was prospective participants’ not returning the screening telephone call. However, despite challenges to recruitment in health-related research, recruitment rates to this study were typical of other clinical trials.^29^ It is possible that participants agreeing to be involved in this study were patients who were more likely to have ongoing health concerns. It is likely that this study population did not include patients who were fully reassured by the assessment process and did not view themselves as having continuing care needs or unanswered questions because such participants may well have thought that they would gain little from being part of the research process. Conducting interviews 1 week postdischarge could influence the content of participant accounts because of events occurring during the recovery period. However, many patients do not process their illness experience until after discharge from the hospital, with concerns becoming apparent only after the acute event. With patients spending less time in the hospital when assessed with the early rule-out pathway, it was believed important to capture how this period may have been influenced by implementation of the early rule-out pathway. We also acknowledge that previous illness experience could affect how participants interpreted information and events during the chest pain assessment process. For some participants, returning to the hospital for a research interview appeared to be used as a further opportunity for contact with a health care practitioner. Although we acknowledge that the study population may represent those who are more concerned about their heart, it is these very patients who require continued support. Potential biases may also exist in selection of participants and coding, although efforts were made to reduce these potential effects. Initial identification of potential participants was performed by nurses from the ED research team, who were not involved with any other aspect of the study and were unaware of the emerging themes of the research. Participant selection could not therefore be influenced by emerging study data. Additionally, because data collection and analysis occurred concurrently, blinding to study group was not possible. All interviews were conducted by A.V.F., although interview technique was discussed and transcripts were reviewed with an experienced senior colleague (S.C.-B.). The grouping of codes into themes was performed through discussion with S.C.-B., including a review of the patient narratives and coding categories. Throughout the process of data collection and analysis, researcher reflexivity was used to discuss any possible biases.

## Discussion

In this study, we explored patient experience of chest pain in 2 groups of patients assessed before and after the implementation of an early rule-out pathway for myocardial infarction. Using individual patient interviews allowed participants to describe their experiences in their own words, thereby providing data that would not be captured with quantitative data collection methods. This study adds to a prospective questionnaire study of patients admitted to a short-stay ward with symptoms of suspected acute coronary syndrome, in which closed questions were posed about the acceptability of an early discharge pathway at patient discharge.^30^ These methods may not capture the richness of experience, not least because many patients do not process their illness experience until after discharge from the hospital, with concerns becoming apparent only after the acute event.

We report 5 major findings. First, it was common for participants to seek help from other health care sources before presentation to the ED. Second, discordance sometimes exists between the objective interpretation of troponin results by clinicians and the ongoing illness episode experienced by patients. Third, pretest information, trust in the clinician, and active listening may enhance reassurance gained from negative test results. These first 3 themes were common to participants assessed with both care pathways. Fourth, other themes appeared to relate to the specific care pathway used; routine care procedures appeared to be a source of frustration for participants requiring admission to the hospital for serial blood sampling. This frustration was not evident with the early rule-out pathway. Fifth, patients assessed with the early rule-out pathway appeared less likely to appraise their future health status; therefore, the rapid rule-out of myocardial infarction in the ED may provide less incentive for patients to use their chest pain presentation as an opportunity to address their future health.

The concepts of discordance and reassurance are linked, with reassurance being more difficult to achieve when discordance is present. Reassurance, diagnosis, explanation, and advice are the main interventions reducing suffering for patients in the ED.^31^ The need for reassurance, and therefore the use of mechanisms for the development of reassurance, emerged as a key theme in this study. We propose a model (Figure 2) in which communication interventions incorporate the provision of pretest information in regard to troponin testing, active listening, and acknowledgment of the patient’s illness concerns. This should lead to the development of trust between the clinician and patient and may aid patients in considering their illness episode as concluded and therefore feeling reassured.

The benefit of pretest information in promoting reassurance is supported by several randomized controlled studies.^32^, ^33^, ^34^ In addition, verbal information has been shown to be more effective than written information in providing reassurance from negative exercise test results for patients with chest pain.^33^ Many studies support the idea that, for patients, a negative test result in itself may not be reassuring.^35^, ^36^, ^37^, ^38^ Patients may be ill prepared for negative or normal findings; therefore, giving information before initial blood sampling about the meaning and subsequent care procedures related to troponin testing may aid in preparing patients for the idea of direct discharge from the ED.

The absence of evidence of active listening was common in accounts of patients who were not reassured by the chest pain assessment process. A shared perspective between the patient and clinician on the cause of symptoms and course of action is positively associated with resolution of symptoms.^39^ This requires effective communication and recognition of the problem as expressed by the patient. Without this, a patient may believe himself or herself ill equipped to manage ongoing symptoms. Achieving a shared understanding can be difficult because clinicians and patients often understand health and illness through different lenses.^40^ This is evident by the clinician’s ability to interpret a low troponin concentration and rule-out of myocardial infarction as a conclusion to the illness episode, which can nonetheless remain very current to the patient. The patient-clinician relationship can be strengthened when patients have the opportunity to express their concerns and the clinician shows empathy with and responds to individual circumstances. When these critical points in communication break down, trust is lost and uncertainty for the patient may prevail. It appeared that patients’ trust in their clinician had to be earned through effective communication. Patients interpret reassurance in the context of their own views and illness perceptions; therefore, a key to successful reassurance is the clinician’s ability to identify and acknowledge the patient’s perspectives in regard to his or her symptoms and related concerns. When there is failure to give credence to the patient’s perspective, or the clinician’s view contradicts the patient’s view, reassurance is difficult to achieve.^41^ When the interventions of pretest information, active listening, and development of trust have been satisfied, patients are more likely to view their illness episode as complete.

The development of reassurance could also be hindered by patient interpretation of routine care procedures. The questionnaire study by Hancock and Carlton^30^ suggested that patients would gain reassurance from being admitted to the hospital, yet the evidence from the study reported here has suggested that admission to the hospital may serve to validate that symptoms may be serious, at least for some patients. Additionally, it was rare for a patient to make the decision to attend the hospital for assessment independently without previous contact with the health service. Prompts by a medical professional allow the patient to negotiate access to care and construct attendance at the ED as an appropriate action. If symptoms have been validated by a health professional in this way, then reassurance from the hospital assessment procedures may be more difficult to achieve.

The risk of implementing early rule-out pathways is that they may focus on process-driven care and outcomes (such as complying with the target to admit, transfer, or discharge patients within 4 hours of attendance at the ED) rather than offering a comprehensive care experience to patients. Although patients may accept that the main aim of an assessment is to exclude a serious illness, care can be considered lacking when it fails to address the patient as a whole. Patients view talking to a professional about their situation as an intervention as important as the delivery of negative test results.^16^ Clinical history taking represents a “process” of care in the ED. The frustration of repeating the symptom presentation story, which was highly prevalent among patients admitted to the hospital for serial troponin-level testing and appeared infrequently among those assessed with the early rule-out pathway, exemplifies further that it is the active listening by the clinician and not simply the opportunity to tell the story that is important to patients.

A further concern about implementing an early rule-out pathway is that the accelerated assessment process may provide less of an opportunity for patients to consider their future health than previous assessment approaches. An episode of acute chest pain can serve as a cue to action for this patient population (although we acknowledge that interview participants revealed only an intention to act). It is possible that the cue to action in the early rule-out pathway is less persuasive because symptoms are dismissed by the ED clinician much more quickly. These interview data are concordant with previous work suggesting that consideration of future health goals appears to be a reactive rather than a proactive process.^42^ A clinical consultation may therefore be enriched by harnessing this cue to action and providing a teachable moment to increase perception of personal risk to future ill health. The content of the consultation may shape the perceived threat of disease or belief in the benefit of lifestyle interventions for the patient. The early rule-out pathway, with its focus on the rapid rule-out of myocardial infarction, may not afford the opportunity for this interaction to develop.

In summary, early rule-out pathways will undoubtedly be of major benefit to health care providers by decreasing unnecessary hospital admissions. Avoiding hospitalization and having fewer health care professionals involved in the assessment process were also viewed positively by patients. The successful implementation of these pathways in the ED will be aided by the addition of simple communication interventions during the chest pain assessment process. Confirmation of the absence of an acute cardiac event may not satisfy the care needs of patients. To enhance the care experience of patients presenting to the ED with symptoms of suspected acute coronary syndrome, focus must remain on the comprehensive assessment and care of the patient, and not solely on the rule-out of myocardial infarction.

## Supplementary Data

Appendix E1 Topic guide Table E1 Additional quotes to illustrate themes.

## References

[1] Goodacre S., Cross E., Arnold J. (2005). The health care burden of acute chest pain. Heart.

[2] Cullen L., Greenslade J., Merollini K. (2015). Cost and outcomes of assessing patients with chest pain in an Australian emergency department. Med J Aust.

[3] Hamm C.W., Bassand J.P., Agewall S. (2011). ESC guidelines for the management of acute coronary syndromes in patients presenting without persistent ST-segment elevation: the Task Force for the Management of Acute Coronary Syndromes (ACS) in Patients Presenting Without Persistent ST-Segment Elevation of the European Society of Cardiology (ESC). Eur Heart J.

[4] National Institute of Clinical Excellence (2010). Chest Pain of Recent Onset: Assessment and Diagnosis of Recent Onset Chest Pain or Discomfort of Suspected Cardiac Origin.

[5] Boyle A., Weber E. (2017). Are rising admission thresholds good medicine?. Emerg Med J.

[6] Roffi M., Patrono C., Collet J.P. (2016). 2015 ESC guidelines for the management of acute coronary syndromes in patients presenting without persistent ST-segment elevation: Task Force for the Management of Acute Coronary Syndromes in Patients Presenting Without Persistent ST-Segment Elevation of the European Society of Cardiology (ESC). Eur Heart J.

[7] National Institute of Clinical Excellence Myocardial infarction (acute): early rule out using high-sensitivity troponin tests (Elecsys Troponin T high-sensitive, ARCHITECT STAT High Sensitive Troponin-I and AccuTnI+3 assays). Diagnostic guidance 15. https://www.nice.org.uk/guidance/dg15/chapter/1-Recommendations.

[8] Rubini Gimenez M., Twerenbold R., Jaeger C. (2015). One-hour rule-in and rule-out of acute myocardial infarction using high-sensitivity cardiac troponin I. Am J Med.

[9] Pickering J.W., Greenslade J.H., Cullen L. (2016). Validation of presentation and 3 h high-sensitivity troponin to rule-in and rule-out acute myocardial infarction. Heart.

[10] Carlton E., Greenslade J., Cullen L. (2016). Evaluation of high-sensitivity cardiac troponin I levels in patients with suspected acute coronary syndrome. JAMA Cardiol.

[11] Bandstein N., Ljung R., Johansson M. (2014). Undetectable high-sensitivity cardiac troponin T level in the emergency department and risk of myocardial infarction. J Am Coll Cardiol.

[12] Body R., Carley S., McDowell G. (2011). Rapid exclusion of acute myocardial infarction in patients with undetectable troponin using a high-sensitivity assay. J Am Coll Cardiol.

[13] Mueller C., Giannitsis E., Christ M. (2016). Multicenter evaluation of a 0-hour/1-hour algorithm in the diagnosis of myocardial infarction with high-sensitivity cardiac troponin T. Ann Emerg Med.

[14] Cullen L., Mueller C., Parsonage W.A. (2013). Validation of high-sensitivity troponin I in a 2-hour diagnostic strategy to assess 30-day outcomes in emergency department patients with possible acute coronary syndrome. J Am Coll Cardiol.

[15] Agard A., Bently L., Herlitz J. (2005). Experiences and concerns among patients being treated for atypical chest pain. Eur J Intern Med.

[16] Jerlock M., Gaston-Johanssen F., Danielson E. (2005). Living with unexplained chest pain. J Clin Nurs.

[17] Johnson M., Goodacre S., Tod A. (2009). Patients' opinions of acute chest pain care: a qualitative evaluation of chest pain units. J Adv Nurs.

[18] Shah A.S.V., Anand A., Strachan F.E. (2018). High-sensitivity troponin in the evaluation of patients with suspected acute coronary syndrome: a stepped-wedge, cluster-randomised controlled trial. Lancet.

[19] Shah A.S.V., Anand A., Sandoval Y. (2015). High-sensitivity cardiac troponin I at presentation in patients with suspected acute coronary syndrome: a cohort study. Lancet.

[20] Chapman A.R., Anand A., Boeddinghaus J. (2017). Comparison of the efficacy and safety of early rule-out pathways for acute myocardial infarction. Circulation.

[21] Chin C.W., Shah A.S., McAllister D.A. (2014). High-sensitivity troponin I concentrations are a marker of an advanced hypertrophic response and adverse outcomes in patients with aortic stenosis. Eur Heart J.

[22] Shah A.S., Griffiths M., Lee K.K. (2015). High sensitivity cardiac troponin and the under-diagnosis of myocardial infarction in women: prospective cohort study. BMJ.

[23] Maritz J., Jooste K. (2011). Debriefing interviews and coaching conversations: strategies to promote student reflexivity and action. S Afr J High Educ.

[24] Rolfe G. (2006). Validity, trustworthiness and rigour: quality and the idea of qualitative research. J Adv Nurs.

[25] Barbour R. (2008). Introducing Qualitative Research: A Student's Guide to the Craft of Doing Qualitative Research.

[26] SAGE Framing a dissertation through a research tradition. https://us.sagepub.com/sites/default/files/upm-binaries/90407_Chapter_3_Framing_a_Dissertation_Study_Through_a_Research_Tradition.pdf.

[27] Braun V., Clarke V. (2006). Using thematic analysis in psychology. Qual Res Psychol.

[28] Upshur R., Morse J., Swanson J., Kuzel A. (2001). The status of qualitative research as evidence. The Nature of Qualitative Evidence.

[29] Gross C., Mallory R., Heiat A. (2002). Reporting the recruitment process in clinical trials: who are these patients and how did they get there?. Ann Intern Med.

[30] Hancock I., Carlton E. (2017). Do low-risk patients with non-cardiac chest pain prefer early discharge after rapid rule-out in the emergency department?. Emerg Med J.

[31] Body R., Kaide E., Kendal S. (2015). Not all suffering is pain: sources of patients' suffering in the emergency department call for improvements in communication from practitioners. Emerg Med J.

[32] Hicks K., Cocks K., Corbacho M. (2014). An intervention to reassure patients about test results in rapid access chest pain clinic: a pilot randomised controlled trial. BMC Cardiovasc Disorders.

[33] Petrie K.J., Muller J.T., Schirmbeck F. (2007). Effect of providing information about normal test results on patients' reassurance: randomised controlled trial. BMJ.

[34] Serinken M., Zencir M., Karcioglu O. (2009). Value of the timing of informing the emergency department patients on cardiac test results: a randomized controlled study. Eur J Emerg Med.

[35] van Ravesteijn H., van Dijk I., Darmon D. (2012). The reassuring value of diagnostic tests: a systematic review. Patient Educ Couns.

[36] Rolfe A., Burton C. (2013). Reassurance after diagnostic testing with a low pretest probability of serious disease: systematic review and meta-analysis. JAMA Intern Med.

[37] Petrie K., Sherriff R. (2014). Normal diagnostic test results do not reassure patients. Evid Based Med.

[38] Rising K.L., Hudgins A., Reigle M. (2016). “I'm just a patient”: fear and uncertainty as drivers of emergency department use in patients with chronic disease. Ann Emerg Med.

[39] Roter D.L. (2002). The enduring and evolving nature of the patient-physician relationship. Patient Educ Couns.

[40] Street R.L., Makoul G., Arora N.K. (2009). How does communication heal? pathways linking clinician-patient communication to health outcomes. Patient Educ Couns.

[41] Donovan J., Blake D. (2000). Qualitative study of interpretation of reassurance among patients attending rheumatology clinics: “just a touch of arthritis, doctor?”. BMJ.

[42] Lawton J. (2002). Colonising the future: temporal perceptions and health relevant behaviours across the lifecourse. Sociol Health III.

