# Supplementary material for: Exploring Patient Experience of Chest Pain Before and After Implementation of an Early Rule-Out Pathway for Myocardial Infarction: A Qualitative Study
Source: Ann Emerg Med. 2020 Apr;75(4):502–13. doi: 10.1016/j.annemergmed.2019.11.012 (PMC7105816; doi:10.1016/j.annemergmed.2019.11.012)
Supplement: Appendix E1 — Topic guide Table E1 Additional quotes to illustrate themes. [file mmc1.docx]

# SUPPLEMENTARY APPENDIX

**Exploring patient experience of chest pain before and after implementation of an early rule-out pathway for myocardial infarction: a qualitative study**

Amy V Ferry PhD,^1^ Fiona E Strachan PhD,^1^ Stacey D Stewart MN,^1^ Lucy Marshall MSc,^1^
Kuan Ken Lee MD,^1^ Atul Anand MD,^1^Anoop SV Shah MD PhD,^1,2^Andrew R Chapman MD,^1^ Nicholas L Mills MD PhD^1,2^ Sarah Cunningham-Burley PhD^2,3^

*^1^BHF Centre for Cardiovascular Science, University of Edinburgh, United Kingdom*

*^2^ Usher Institute of Population Health Science and Informatics, University of Edinburgh, United Kingdom*

*^3^Centre for Biomedicine, Self and Society*, *University of Edinburgh, United Kingdom*

**Correspondence and requests for reprints:**
Amy Ferry

BHF Centre for Cardiovascular Science

SU305 Chancellors Building

Royal Infirmary of Edinburgh

Edinburgh EH16 4SA

United Kingdom

Tel: +44-131-242-6515

Fax: +44-131-242-6379

Email: [Amy.Ferry@ed.ac.uk](mailto:Amy.Ferry@ed.ac.uk)

**Appendix E1 Topic guide**

**An exploration of the lived experience of chest pain and how it may be shaped by the implementation of an early rule-out pathway for myocardial infarction**

**Topic guide**

*1) Patients’ beliefs of the cause of chest pain*

*2) The interrelationship between health care seeking behaviour, self-care and social support*

*3) Patients’ experiences of how a cardiac cause has been ruled out*

*4) Patients’ experience of standard of care*

As the interview progresses the topic guide will be used flexibly. The participant will determine the direction of conversation. Prompts are listed below to facilitate the interview.

**Introduction**

Revisit aim of study and introduce researcher

Discuss ethical issues – consent, confidentiality, anonymity, permission to record

Talk through and gain informed consent

- 1. ***Patients’ beliefs of the cause of chest pain***

Explore the most recent visit to hospital with chest pain

**Could you tell me about what happened to take you in to hospital last week?**

- In your own words what do you think caused the pain?

- 1. ***The interrelationship between health care seeking behaviour, self-care and social support***

**Could you explain what had been happening before going in to hospital?**

- GP visit/NHS 24/lay support sought/internet advice

- sought medical advice for chest pain on previous occasions

- social support available

**How are you getting on now you have been home for a week or so?**

- mentions anxieties/worries - can you tell me more about that

- returning to normal activities or not? If not, expand on the cause.

- 1. ***Patients’ experiences of how a cardiac cause has been ruled out***

**Tell me about the tests that you had done, - what were the doctors looking for?**

**How did you feel during this time?**

- Are you reassured by what you have been told regarding your symptoms?

- Is there any kind of treatment/interaction that would have made you feel better/led you to believe that your symptoms were not cardiac?

- 1. ***Patients’ experience of standard of care***

**Can you tell me about how you felt when you were told you could go home?**

- What did you think about the communication provided by staff regarding your symptoms and potential causes?

- mentions relationships with healthcare staff - expand

- Was there any particular interaction that you found useful?

- How did you feel on discharge? (thoughts/concerns/emotions/plans/aims)

- mentions emotional responses - expand

- mentions return to work or normal activities if not already covered

- did they feel they knew what they could or couldn’t do on going home?

- have they used the health service since discharge due to unanswered questions?

- Is there anything that would have improved the way that you received care and information regarding your symptoms?

- 1. ***Future health (added due to emergent theme)***

***Has your episode of pain altered the way think about anything or the way you would act in a particular situation?***

- Introduces the topic of health behaviours – expand
- Introduces future risk of heart disease – explore
- Introduces health promotion activity

**Closing the interview**

Is there anything we haven’t talked about that you would like to cover?

Is there anything you would like to ask me before we end?

Is there any message you would like to give the NHS about attending the ED for assessment of chest pain?

Thank you

**Table E1 Additional quotes to illustrate themes**

| **Theme** | **Evidence** | **Participant** |
| --- | --- | --- |
| **Help seeking behaviour** | P: You…well, you, you don’t want to time waste in any way, because…  I: Yeah.  P: …I mean, you appreciate these services when you really need them, and, and you certainly don’t want to…and you hear all, all the publicity about how stressed they are, and how they’re not meeting the target, and all that…  I: Hmm.  P: …and you feel so sorry, be…and the last thing you want to do is to, is to waste time | Participant 3, >65 female (pre-implementation) |
|  | P: But I was really, you're really hesitant to phone 999, although everybody has told me, that's what you do. But you're fr...frightened to do it, in case it's not anything, and you've wasted their time.  I: Right.  P: That's how you feel. Maybe this generation feels like that. | Participant 31, >65 female (post-implementation) |
|  | P: I was a bit scared. I said, because…I think it’s…when the doctor telt me, phone nine nine nine, I’m, oh my God…  I: Mm.  P: Yeah, I mean…I wasn’t looking for that. I thought he was going to say, well up your aspirin, or, do this, or, do that. But, no, dial nine nine nine… | Participant 18, >65 male (pre-implementation) |
|  | P: The more I sat in the doctors surgery the more...I think as soon as the doctor started to hum, not hum and haw, but he was writing a few things and I could see him writing a few things, I thought, well hold on a minute. They're taking this more seriously than I was thinking. | Participant 33, ≤65 male (post-implementation) |
| **Discord** | P: I'm still a bit apprehensive about...I just wonder when's this next attack going to come  on? Is it going to last as long? Is it going to be as sore? | Participant 10, ≤65 male (pre-implementation) |
|  | P: when I left, obviously I was, erm, very happy that I didn't suffer a heart attack. Erm, but I knew there was something wrong, there's still something wrong. There's something there, you don't just have those feelings and stuff, you know your body is trying to tell you something. | Participant 11, ≤65 male (pre-implementation) |
|  | P: that's part of the frustration…it’s when somebody turns round to you and says, oh it's probably not cardiac…  I: Uh-huh.  P: …and you're thinking, I'm the one that's had the heart attack I know…  I: Yes.  P: …when there is something cardiac wrong with me, I know. | Participant 13, ≤65 female (pre-implementation) |
|  | P: They weren’t really sure as I say, they said the ECG come back clear, the blood came back clear, but they think it's this spasm, but it, they never really said it is, it's definitely not a heart attack and it is definitely the spasm, so, I don't know where I am. | Participant 44, >65 female (post-implementation) |
| **Reassurance** | P: The doctor came along and he explained that, you know, there were many things it could be, because there's lots of things that mimic the symptoms of a heart attack. Erm, but they can't rule out, obviously until they've had the tests back.  I: Yeah.  P: Erm, and I found that helpful, because I felt, I felt he was being honest, he wasn't sort of...  I: Okay.  P: ...you know, hiding behind anything. So that was useful. Erm, and then, yeah, I had the first blood test, that came back okay, and then I was told I had to have another one in six hours' time, or whatever. I didn't feel that I'd been forgotten or anything, which I think is important. Erm, and then, when they came to, they said, [name], we can discharge you. The consultant explained that, from the blood tests, that it, you know, they could confirm that it wasn't any form of heart attack. They thought that it possibly was something called esophageal spasm. | Participant 16, ≤65 female (pre-implementation) |
|  | I: What actually gives you that reassurance?  P: It's just the, the know...the knowledge they're giving you.  I: Uh-huh.  P: Once you have that knowledge, then you can sort it out in your own mind.  I: Right.  P: Well it's this, it's this explaining everything.  I: Yeah, yeah.  P: If they don't explain things, there's no way you can feel better.  I: No.  P: Because there's always gonna be something nagging in the back of your mind. But if it's explained to you properly...unless you're thinking on a different planet, you're bound to process it. It's only if, I think it's, if you don't have the right questions to ask them...  I: Yes. P: ...but if the questions you do ask them are answered, and answered properly, then you're bound to feel reassured. And I think they deal with it pretty well. | Participant 21, ≤65 male (pre-implementation) |
|  | P: This time I’ve got to say I think the doctor in particular – whose name I can’t recall – um…with all… The…the first nurse I met, um, was very reassuring, um [pause], then the doctor I was involved with, eh, came and explained stuff very well. In fact I had to have…I had to have a second blood test because the first blood test had been taken inside the three hour window, you know…  I: Oh, okay, yeah, yeah.  P: …so I had to wait around a bit, and the…the nurse made sure that the doctor was aware when my second result came back. It just felt…and the unit was heaving that day as well. So I think, um [pause], the multi-faceted element is, was somebody had bothered to explain that, and I’ve never heard from Aberdeen that actually they did this test and it was clear, or not. | Participant 41, ≤65 male (post-implementation) |
|  | P: Um, basically he told me about that, er, all the, you know, blood tests and including the cardiac enzyme they are all basically, um, came fine and, er, 12 lead ECGs is fine, the chest x-ray is fine and they can't basically specifically pinpoint any reason. So they don't think that any of the cardiac and all that and, and I was fine with that and I said that so I'm going to get discharged and he said, oh yes, are you happy with that? And I said that, oh yeah, absolutely. Because at the end of the day if I don't need to stay in the hospital if the, all the basic tests are fine, then, then that’s fine, that’s a big relief, so that was like a short conversation but very to the point and precise which was good. | Participant 43, ≤65 female (post-implementation) |
| **Influence of ED routines** | P: …I didn't think there was any erm, communication at all. They were only…  I: Right okay.  P: …interested in, in getting this blood out and sending this blood away and out the door. There wasn't any…there wasn't any… As, as a person you weren't kind of treated like a person, you were more treated like oh well, let's see what's going on. You've got a pain in your chest, right, get that blood out, do that test and out. | Participant 39, >65 female (post-implementation) |
|  | P: I’m not sure which part of it made me feel confident I was being looked after but the process itself was really quite reassuring that, yes, I was feeling ill, I was reassured that I was not dying [laugh], not in any direct way, but in a subliminal way, there was no panic, there was no concern. They were all very professional and very calm. And that really helped me…  I: Okay.  P: …to be…to be honest, because if they’re not concerned about emergency procedures and…  I: Yep.  P: …sticking tubes down your throats and doing…  I: Yep.  P: …er, CPR, then…and that would be an excitement – in inverted commas – for them, but it wasn’t where we were at.  I: Yep.  P And I felt that that helped me… | Participant 37, >65 male (post-implementation) |
|  | P: The nurses and the support workers didn't really say anything, they just came in and they just went, they went about their business taking whatever they needed to take and...but that obviously, there’s obviously some kind of process and procedure or protocol that they follow, so knowing that it is potential chest or cardiac then obviously things kick into action...  I: Yes.  P: ...because it was pretty, it was pretty seamless when I, when I went in. | Participant 24, ≤65 male (post-implementation) |
|  | P: The only thing that got, got quite tiring was, I seemed to be telling the same story…  I: Yes.  P: …over, and over and over again. But I, you know, you felt, well, are they checking to see if they can catch you out, and you change your mind?  I: Yes.  P: Which I didn’t really believe…  I: Yeah.  P: …but you, you began, in the course of the morning after, you know, several hours, and people were still asking you, you know, what was…how did this start, and all the rest of it: I thought, oh my…I was really getting to the point where I didn’t want to, to, er, tell it again. | Participant 2, >65 female (pre-implementation) |
|  | P: The second time they did it, I was a bit more concerned that they’d actually found something on the first trace…  I: Ah okay.  P: …and they said…they didn’t explain it then. [Cough] That’s not a criticism.  I: Yeah.  P: I wasn’t apprehensive in any way because I felt at that time I was getting sucked in to being looked after by the health service professionals and that they were going to be managing my visit through, er, A&E. So that was how I felt at that time, so…  I: Right. But the fact that they repeated that test…  P: That was a concern…  I: Right.  P: …because in health…if you have a test result that you’re not sure of or abnormal…  I: Okay.  P: …you confirm that that’s the case.  I: Yeah. Yeah.  P: You don’t confirm normal tests… | Participant 37, >65 male (post-implementation) |
| **Approaches to future health** | P: I could see my blood pressure, and it had come down a bit, but it wasn't down to normal.  I: Uh-huh.  P: And I think that's another thing that I, perhaps, don't feel fully, erm, explained, is why did my blood pressure go up like that. And, you know, if it was just something like the esophageal spasm, or whatever, well why, why did my blood pressure...you know, was that just because of the anxiety.  I: Uh-huh.  P: Can anxiety push your blood pressure up like that, and not your pulse. My pulse was less than fifty.  I: Right.  P: You know, so that makes me think, oh gosh, you know, have I got a blood pressure problem now. Erm, so yeah, I, I don't know whether I still feel I should go to my doctor, and even though a letter has gone to them. | Participant 16, ≤65 female (pre-implementation) |
|  | I: So has it made you kind of address anything?  P: Made me think about my smoking status.  I: Right.  P: Erm, and I'm think right, I really need to stop. I haven't actually completely stopped, but I probably…I don't smoke a lot anyway, so. I think… It's made me feel better that I know there's nothing wrong with my heart, and I'm not, I'm not… Like before this happened there have been times when I've thought oh, you know, this smoking's going to take its toll at some point in my life, or you know, having this wine's going to take its toll. And you know that when you get older you don't change things, things are gonna happen, you know, and not for the better. But erm, it's not radically changed my lifestyle. But I'm still stopped drinking Monday to Friday. | Participant 30, ≤65 female (post-implementation) |
|  | P: I suppose, it's really left me thinking that it could happen, a, a heart attack could happen to anybody, at any age.  I: Right.  P: And I think if you're, obviously, if you're so stressed about things, it's gonna increase your risk, a lot. So that's, yeah, I've definitely thought about that since I've come home.  I: Have you?  P: Yeah.  I: Yeah.  P: Trying to think of, like, de-stressing myself, and you know, you only live once, kind of thing.  I: Yeah.  P: And trying to take...I haven't, as I say, I haven't actually been at my work up there, it's just round the corner. Erm, I haven't been up there, but just trying to think of ways, when I do go back, to try and take a step back, for my own, my own health. | Participant 48, ≤65 female (post-implementation) |
